# Inferior parietal cortex represents relational structures for explicit transitive inference

**DOI:** 10.1093/cercor/bhae137

**Published:** 2024-04-05

**Authors:** Biman Xu, Jing Wu, Haoyun Xiao, Thomas F Münte, Zheng Ye

**Affiliations:** Institute of Neuroscience, Center for Excellence in Brain Science and Intelligence Technology, Chinese Academy of Sciences, Yueyang Road 320, Shanghai 200031, China; University of Chinese Academy of Sciences, Yanqihu East Road 1, Beijing 101408, China; Institute of Neuroscience, Center for Excellence in Brain Science and Intelligence Technology, Chinese Academy of Sciences, Yueyang Road 320, Shanghai 200031, China; University of Chinese Academy of Sciences, Yanqihu East Road 1, Beijing 101408, China; Institute of Neuroscience, Center for Excellence in Brain Science and Intelligence Technology, Chinese Academy of Sciences, Yueyang Road 320, Shanghai 200031, China; University of Chinese Academy of Sciences, Yanqihu East Road 1, Beijing 101408, China; Center for Brain, Behavior & Metabolism, University of Lübeck, Ratzeburger Allee 160, Lübeck 23562, Germany; Institute of Neuroscience, Center for Excellence in Brain Science and Intelligence Technology, Chinese Academy of Sciences, Yueyang Road 320, Shanghai 200031, China

**Keywords:** logical reasoning, transitive inference, relational structure, inferior parietal cortex, fMRI

## Abstract

The human brain is distinguished by its ability to perform explicit logical reasoning like transitive inference. This study investigated the functional role of the inferior parietal cortex in transitive inference with functional MRI. Participants viewed premises describing abstract relations among items. They accurately recalled the relationship between old pairs of items, effectively inferred the relationship between new pairs of items, and discriminated between true and false relationships for new pairs. First, the inferior parietal cortex, but not the hippocampus or lateral prefrontal cortex, was associated with transitive inference. The inferior parietal activity and functional connectivity were modulated by inference (new versus old pairs) and discrimination (true versus false pairs). Moreover, the new/old and true/false pairs were decodable from the inferior parietal representation. Second, the inferior parietal cortex represented an integrated relational structure (ordered and directed series). The inferior parietal activity was modulated by serial position (larger end versus center pairs). The inferior parietal representation was modulated by symbolic distance (adjacent versus distant pairs) and direction (preceding versus following pairs). It suggests that the inferior parietal cortex may flexibly integrate observed relations into a relational structure and use the relational structure to infer unobserved relations and discriminate between true and false relations.

## Introduction

The human brain is distinguished by its ability to perform explicit logical reasoning like transitive inference. When told “Adele is taller than Billie” and “Taylor is taller than Adele,” one might explicitly construct a relational structure that “Taylor>Adele>Billie” and logically infer that “Taylor is taller than Billie” even though the information is not directly given. Nonhuman primates do not always exhibit transitive-inference-like behavior, even after extensive training (Gillan 1981). Building machines that make efficient inferences like humans remains a grand challenge in artificial intelligence research (Lake et al. 2017).

In previous studies, however, transitive inference has often been conceptualized as an implicit process relying on reinforcement learning (Bryant and Trabasso 1971; McGonigle and Chalmers 1977; Vasconcelos 2008). Implicit transitive inference engages a distributed brain network comprising the hippocampus, lateral prefrontal cortex, and parietal cortex (Acuna et al. 2002; Van Opstal et al. 2008; Van Opstal et al. 2009; Zalesak and Heckers 2009; Hinton et al. 2010; Zhang et al. 2022). Frank et al. (2005, 2006) questioned the assumption that implicit transitive inference uses the same system as human explicit logical reasoning (see discussion). Their work suggests that explicit and implicit transitive inference are dissociable at behavioral and neural levels.

**Fig. 1:**
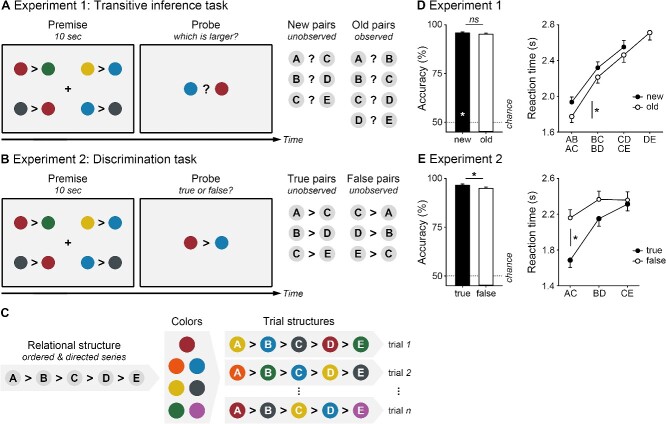
Tasks and performances. Participants viewed four randomized premises and were asked to **A**) infer the relationship between a new pair of items, recall the relationship between an old pair of items (experiment 1), or **B**) judge whether the given relationship between a new pair of items was true or false following the premises (experiment 2). **C**) The underlying relational structure was always an ordered and directed series, but the exact relational structure varied from trial to trial. Means and SEMs of the accuracy and reaction time **D**) for new and old pairs (experiment 1) and **E**) for true and false pairs (experiment 2). Asterisks, *P* < 0.05; ns, not significant.

In this study, we conceptualize transitive inference as an explicit logical reasoning process in working memory and revisit its neural basis in two functional MRI (fMRI) experiments. In experiment 1, participants viewed four randomized premises describing the “larger than” relation among five items. They were then asked to infer the relationship between a new pair of items they had never observed or recall the relationship between an old pair of items they had observed (Fig. 1A). In experiment 2, they were asked to judge whether the given relationship between a new pair of items was true or false following the premises (Fig. 1B). The underlying relational structure was always an ordered and directed series (Fig. 1C). However, the exact relational structure varied from trial to trial. Participants had to construct the relational structure flexibly in every trial rather than stick to a fixed structure for the whole experiment.

**Fig. 2:**
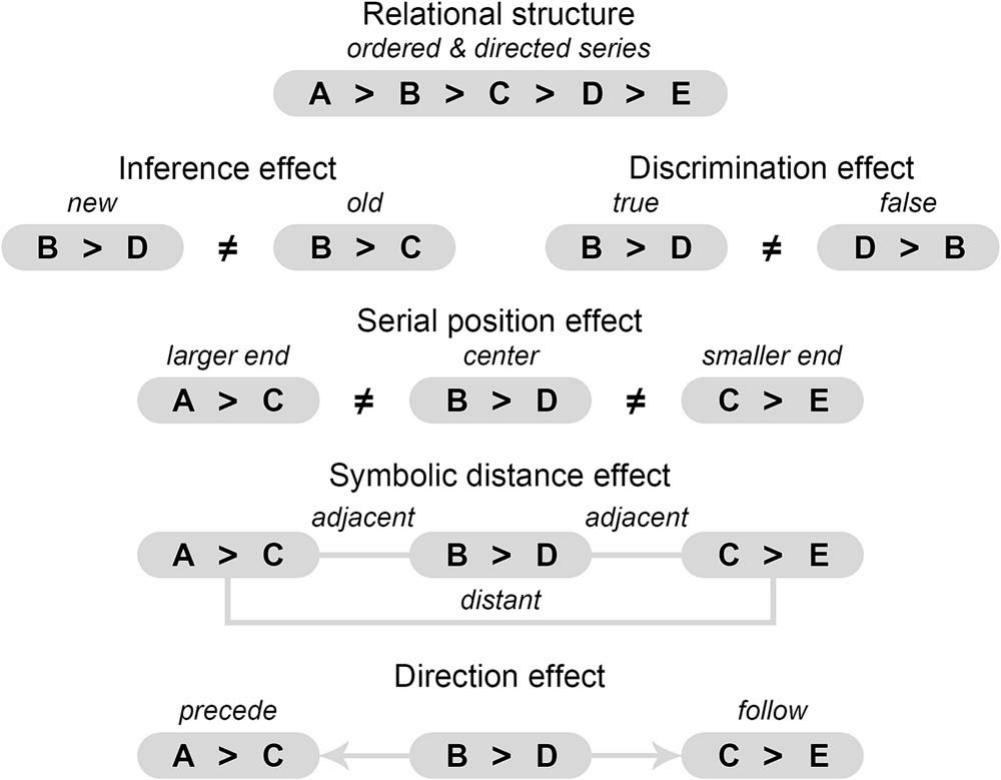
Definition of effects and examples.

Previous studies on the neural representation of structural knowledge and transitive inference directed our attention to the parietal cortex, lateral prefrontal cortex, and hippocampus (Wendelken and Bunge 2010; Backus et al. 2016; Alfred et al. 2018; Luyckx et al. 2019; Pudhiyidath et al. 2022; Xie et al. 2022; Nelli et al. 2023). Suppose a region serves to integrate observed relations of items into a relational structure (ordered and directed series) and uses the relational structure to infer unobserved relations of items. In that case, we should observe the following five effects (Fig. 2). First, the region should distinguish not only between observed and unobserved relations (inference effect, new versus old pairs, e.g. [B > D] ≠ [B > C]) but also between unobserved relations consistent with and those inconsistent with the series (discrimination effect, true versus false pairs, e.g. [B > D] ≠ [D > B]). Second, the region should distinguish between observed/unobserved relations near the ends and those near the center of the series (serial position effect, larger/smaller end versus center pairs, e.g. [A > C] ≠ [B > D] ≠ [C > E]) (Crowder 1976; Woocher et al. 1978). Third, the region should distinguish between observed/unobserved relations adjacent to each other and those distant from each other (symbolic distance effect, adjacent versus distant pairs, e.g. for [A > C], [B > D] is an adjacent pair and [C > E] is a distant pair). The serial position and symbolic distance effects at the relation level are analogous to but different from those at the item level. Finally, for a given observed/unobserved relation, the region should distinguish between observed/unobserved relations preceding it and those following it (direction effect, preceding versus following pairs, e.g. for [B > D], [A > C] is a preceding pair and [C > E] is a following pair) (Ma et al. 2019). The inference and discrimination effects link the region with transitive inference but do not tell whether or how the relational structure is constructed. The serial position, symbolic distance, and direction effects reflect characteristics of the relational structures. If the above effects occur in the same region, then this region likely represents the integrated relational structure for explicit transitive inference.

## Experiment 1: transitive inference task

### Materials and methods

The study was approved by the ethics committee of the Chinese Academy of Sciences Institute of Neuroscience following the Declaration of Helsinki. All participants signed written informed consent before participating in this study.

#### Participants

Thirty healthy adults participated in experiment 1 (19 women, age 23.9 ± 1.3 years, education 16.9 ± 1.3 years). All participants were right-handed and had normal or corrected-to-normal vision. They had no history of neurological or psychiatric diseases. Five additional participants were excluded because they did not complete MRI scanning (*n* = 2) or had excessive head motion (*n* = 3).

#### Experimental design and procedure

All participants completed the transitive inference task with fMRI scanning (50–60 min, Fig. 1A). In each trial, participants viewed four randomized premises describing the “larger than” relation among five items (10 s). They were asked to infer the relationship between a new pair of items or recall the relationship between an old pair of items (maximum 5 s). The underlying relational structure was always an ordered and directed series. The trial structure varied, with items randomly selected from a color pool and their relations arbitrarily assigned (Fig. 1C). Participants responded by pressing left/right buttons with their right hand. No feedback was given. Each participant completed eight experimental blocks, 16 trials per block (eight new pairs, eight old pairs).

#### Statistical analysis of transitive inference task performance

We controlled behavioral data quality by monitoring premature (reaction time, RT, < 0.1 s) and inattentive responses (RT > 3 SDs above the individual mean). Participants made no premature responses and very few inattentive responses (0.2 ± 0.4%). The inattentive responses were excluded from further analysis.

First, we examined whether participants made transitive inferences effectively using a one-sample *t*-test (new > chance, *P* < 0.05) and a paired-sample *t*-test (new versus old, *P* < 0.05). Second, we examined whether the discriminability of larger and smaller items (*d*′ = *z*_H_ − *z*_FA_, where *z*_H_ and *z*_FA_ indicate the *z* scores of the hits and false alarms, respectively) (MacMillan 1993) was modulated by inference using a paired-sample *t*-test (new versus old, *P* < 0.05). Third, we examined whether the RT was modulated by inference and serial position using an ANOVA with two factors (*P* < 0.05), Pair (new, old) and Position (AC/AB, BD/BC, CE/CD).

#### Acquisition of MRI and fMRI data

MRI/fMRI data were acquired on a Siemens Tim Trio 3-T MRI scanner with a 32-channel head coil. Structural T1-weighted images used a magnetization-prepared rapid gradient-echo sequence (192 sequential sagittal slices, repetition time 2,300 ms, echo time 3 ms, flip angle 9°, field of view 256 × 256 mm^2^, and spatial resolution 1 × 1 × 1 mm^3^). Functional T2*-weighted images used a simultaneous multislice echo-planar imaging sequence with acceleration factor 2 (46 interleaved axial slices, repetition time 1,500 ms, echo time 30 ms, flip angle 60°, field of view 220 × 220 mm^2^, and spatial resolution 3 × 3 × 3 mm^3^). Field map images used a gradient-echo sequence (46 axial slices, repetition time 500 ms, short echo time 4.92 ms, long echo time 7.38 ms, flip angle 60°, field of view 220 × 220 mm^2^, and spatial resolution 3 × 3 × 3 mm^3^).

#### Preprocessing of fMRI data

Functional MRI data were preprocessed with SPM12 (v7771, www.fil.ion.ucl.ac.uk/spm). A voxel displacement map was derived from the presubtracted phase and magnitude field map images for correcting geometric distortion. The first five images of each block were discarded to allow magnetization equilibration. Other images were corrected for slice acquisition time difference, realigned to the first image, corrected for geometric distortion, registered to the structural T1-weighted image, normalized to the Montreal Neurological Institute coordinate system, smoothed with a Gaussian kernel, and filtered with a 128-s high-pass filter. For the univariate and psychophysiological interaction (PPI) analyses, the Gaussian kernel was 6-mm full-width half-maximum. For the multivariate decoding and representational similarity analyses, the Gaussian kernel was 4-mm full-width half-maximum.

We controlled fMRI data quality by monitoring the scan-to-scan total displacement (Wilke 2012) (all blocks < 1 mm, 0.36 ± 0.15 mm), field map correction, and spatial normalization (visual inspection).

#### Univariate analysis of the inference and serial position effects

First, we examined whether the parietal, lateral prefrontal, or hippocampal activity was modulated by inference or serial position. The first general linear model (GLM) with canonical hemodynamic response function was built at the subject level, including three new-pair and four old-pair regressors (correct trials only). The premises, incorrect/inattentive trials, and total displacement were included as nuisance regressors. Classical parameter estimation was applied with a one-lag autoregressive model. The univariate inference contrast was old > new pairs. The univariate serial position contrasts were larger-end > center pairs and smaller-end > center pairs. For new pairs, AC was the larger end, BD was the center, and CE was the smaller end. For old pairs, AB was the larger end, BC and CD were the center, and CE was the smaller end. All contrasts were entered into whole-brain one-sample *t*-tests at the group level (voxel-level *P* < 0.001, cluster-level *P* < 0.05 familywise error correction). Results corrected with alternative cluster-based methods are shown as supplemental information.

Second, we visualized the temporal dynamics of the significant univariate inference and serial position effects. Finite impulse response (FIR) timeseries of each probe were extracted from regions of interest (ROIs) defined by the univariate inference and serial position contrasts (see results). For each hemisphere, the percent signal change of ten scans following the probe (0–15 s) was calculated and entered into paired-sample *t*-tests (inference: old > new, serial position: larger end > center, *P* < 0.05 Bonferroni correction).

#### Psychophysiological interaction analysis of the inference effect

First, we examined whether the inferior parietal functional connectivity was modulated by inference. Raw fMRI signals of the left (sphere with peak [−39, −69, 45] and 5-voxel radius) and right inferior parietal seeds (sphere with peak [54, −57, 36] and 5-voxel radius) were demeaned and deconvolved to create PPI variables. The second GLM was built at the subject level, including a PPI regressor, a physiological signal regressor, and an inference contrast regressor. The total displacement was included as a nuisance regressor. The PPI inference contrast was defined as new > old pairs and entered into whole-brain one-sample *t*-tests at the group level (voxel-level *P* < 0.001, cluster-level *P* < 0.05 familywise error correction).

Second, we examined whether the RT inference effect correlated with the left- or right-seed PPI inference effect using forward stepwise regression (*P* < 0.05). The RT inference effect was the mean RT difference between new and old pairs, normalized to the RT of old pairs.

#### Multivariate decoding analysis of pair type

We examined whether the new/old pairs were decodable from the inferior parietal representation. The third GLM was built at the subject level, with each probe as a separate regressor. The premises and total displacement were included as nuisance regressors. Beta values of correct probes were extracted from a series of ROIs defined by the univariate inference contrast (2^n^ voxels around the peak, n≧3) and entered into support vector machines (binary classifiers). The classifier was optimized with a grid-search algorithm of the LIBSVM toolbox (Chang and Lin 2011). For each hemisphere, the decoding accuracy was assessed using 5-fold cross-validation and entered into one-sample *t*-tests (real accuracy > baseline, *P* < 0.05 Bonferroni correction). The baseline accuracy was estimated using the optimized classifier with shuffled data (500 iterations per subject) and averaged across subjects.

#### Representational similarity analysis of the symbolic distance and direction effects

We examined whether the inferior parietal representational similarity between pairs was modulated by symbolic distance and direction. Figure 4B shows the definition of symbolic distance, direction, and ANOVA. In the first GLM, beta values of correct probes were extracted from the left (606 voxels) and right ROIs (614 voxels) defined by the univariate serial position contrast. Pearson correlation coefficients were calculated between pairs, normalized using Fisher’s transformation, and entered into three ANOVAs. The first ANOVA detected the symbolic distance effect with three factors (*P* < 0.05), Distance (1, 2), Pair (new, old), and Hemisphere (left, right). The second ANOVA detected the direction effect with three factors (*P* < 0.05), Direction (preceding/−, following/+), Pair (new, old), and Hemisphere (left, right). The third ANOVA confirmed the direction effect across pair types with two factors (*P* < 0.05), Direction (preceding/−, following/+) and Hemisphere (left, right).

**Fig. 3:**
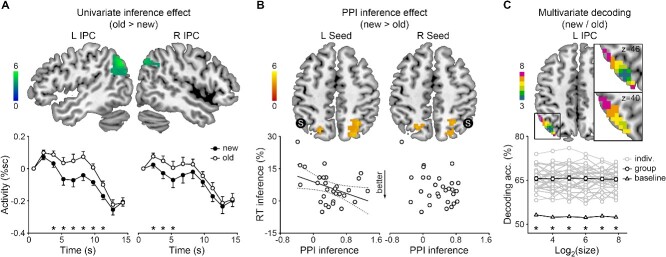
Inference effects in the inferior parietal cortex. **A**) Top: Inference effects on the regional activity of the left and right inferior parietal cortex (L/R IPC). The color scale indicates *t* values. Bottom: Mean FIR timeseries and SEMs of new and old pairs in the corresponding region. %sc, percent signal change; asterisks, *P* < 0.05 corrected. **B**) Top: Inference effects on the PPI of the left and right IPC seeds (S). The color scale indicates *t* values. Bottom: The RT inference effect correlated with the left-IPC PPI. Solid line, *P* < 0.05; dotted lines, 95% confidence intervals. **C**) Top: Left-IPC ROIs of variable sizes. The color scale indicates log_2_(size). Bottom: Individual data, group means, and SEMs of the decoding accuracy of the left-IPC classifiers. Asterisk, *P* < 0.05 corrected.

**Fig. 4:**
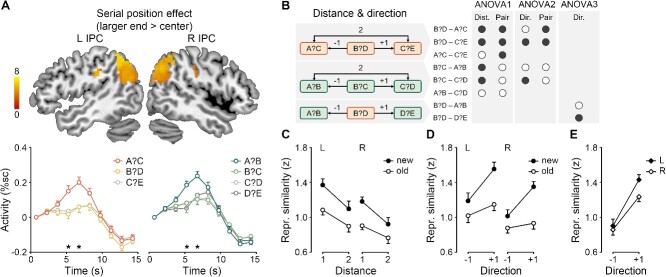
Serial position effect, symbolic distance effect, and direction effect in the inferior parietal cortex. **A**) Top: Serial position effects on the regional activity of the left and right inferior parietal cortex (L/R IPC). The color scale indicates *t* values. Bottom: Mean FIR timeseries and SEMs of new and old pairs in the left IPC. %sc, percent signal change; asterisks, *P* < 0.05 corrected. **B**) Definitions of the symbolic distance, direction, and ANOVA. The hemisphere factor is not shown. Mean inferior parietal representational similarity and SEMs with variable **C**) symbolic distance (ANOVA 1) and **D**–**E**) direction (ANOVA 2–3).

### Results

#### Transitive inference task performance

Participants made transitive inferences effectively (Fig. 1D). The accuracy of new pairs was significantly above chance (one-sample *t*-test, *t*(29) = 82.55, *P* < 0.001), not different from that of old pairs (paired-sample *t*-test, *P* = 0.21). The *d*′ of larger and smaller items was comparable between new (3.56 ± 0.58) and old pairs (3.41 ± 0.49, paired-sample *t*-test, *P* = 0.19).

Participants were slower for new than old pairs. The RT increased as the serial position changed regardless of the pair type. The ANOVA revealed the main effects of Pair (*F*(1,29) = 21.47, *P* < 0.001, *η_p_*^2^ = 0.43) and Position (*F*(2,58) = 107.68, *P* < 0.001, *η_p_*^2^ = 0.79), but no interaction (*P* = 0.28).

#### Inference effects on inferior parietal activity

The inferior parietal activity, but not the hippocampal or lateral prefrontal activity, was modulated by inference (Fig. 3A, Supplemental Figs. S1–S2). The bilateral inferior parietal regions were more activated for old than new pairs (whole-brain one-sample *t*-test, left: peak [−39, −69, 45], *t* = 5.92, 229 voxels, right: peak [54, −57, 36], *t* = 4.69, 65 voxels). No significant effect was obtained for the reverse contrast. The univariate inference effect occurred between 4.5 and 12 s following the probe in the left hemisphere (paired-sample *t*-test, *t*(29) = 3.18–5.02, *P*s < 0.002) and between 3 and 6 s in the right hemisphere (*t*(29) = 3.32–3.82, *P*s < 0.001). Therefore, we focused on the inferior parietal cortex in further analyses.

#### Inference effects on inferior parietal functional connectivity

The inferior parietal functional connectivity was modulated by inference (Fig. 3B). The PPI between the left inferior parietal seed and bilateral superior parietal regions (whole-brain one-sample *t*-test, left: peak [−24, −84, 30], *t* = 4.96, 114 voxels, right: peak [30, −60, 54], *t* = 4.81, 162 voxels) and that between the right inferior parietal seed and bilateral superior parietal regions were enhanced for new than old pairs (left: peak [−21, −66, 57], *t* = 4.42, 45 voxels, right: peak [24, −57, 57], *t* = 5.15, 128 voxels). No significant effect was obtained for the reverse contrast.

The normalized RT inference effect correlated with the left-seed PPI inference effect. The forward stepwise regression model for the RT inference effect (*F*(1,29) = 5.00, *P* = 0.03, *R*^2^ = 0.15) included the left-seed (*t* = −2.24, *P* = 0.03) but not the right-seed PPI inference effect (*P* = 0.95).

#### Inferior parietal representations of new/old pairs

The new/old pairs were successfully decoded from the inferior parietal representation (Fig. 3C). The decoding accuracy of the inferior parietal classifier was above baseline regardless of the ROI size and hemisphere (left: 8–229 voxels, *t*(29) = 17.04–20.97, *P*s < 0.001, right: 8–65 voxels, *t*(29) = 17.61–19.18, *P*s < 0.001).

#### Serial position effects on inferior parietal activity

The inferior parietal activity was modulated by serial position (Fig. 4A, Supplemental Fig. S1). The bilateral inferior parietal regions were more activated for new/old pairs near the larger end than those near the center of the series (whole-brain one-sample *t*-test, left: peak [−54, −51, 39], *t* = 8.14, 606 voxels, right: peak [57, −51, 33], *t* = 7.46, 614 voxels). For new pairs, the univariate serial position effect occurred between 6 and 7.5 s following the probe bilaterally (paired-sample *t*-test, left: *t*(29) = 4.47–4.59, *P*s < 0.001, right: *t*(29) = 4.80–5.86, *P*s < 0.001). A similar pattern was obtained for old pairs (left: *t*(29) = 4.81–5.90, *P*s < 0.001, right: *t*(29) = 4.58–5.00, *P*s < 0.001). However, no significant effect was obtained for the smaller end > center contrast.

#### Symbolic distance and direction effects on inferior parietal representations

The inferior parietal representational similarity between pairs was modulated by symbolic distance and direction. The first ANOVA revealed the symbolic distance effect (Fig. 4C), showing the main effects of Distance (*F*(1,29) = 36.11, *P* < 0.001, *η_p_*^2^ = 0.56), Pair (*F*(1,29) = 12.38, *P* = 0.001, *η_p_*^2^ = 0.30), and Hemisphere (*F*(1,29) = 10.84, *P* = 0.003, *η_p_*^2^ = 0.27), but no interaction (*P*s > 0.11). The inferior parietal representation of a given pair (e.g. A?C) was more similar to that of pairs adjacent to it (e.g. B?D) than that of pairs distant from it (e.g. C?E), regardless of the pair type. New pairs were more similar than old pairs in the inferior parietal representation, probably due to overlapping items.

The second ANOVA revealed the direction effect (Fig. 4D), showing the main effects of Direction (*F*(1,29) = 8.88, *P* = 0.006, *η_p_*^2^ = 0.23), Pair (*F*(1, 29) = 24.66, *P* < 0.001, *η_p_*^2^ = 0.46), and Hemisphere (*F*(1,29) = 11.77, *P* = 0.002, *η_p_*^2^ = 0.29), and interaction of Direction and Pair (*F*(1,29) = 10.96, *P* = 0.002, *η_p_*^2^ = 0.27). The inferior parietal representation of a given pair (e.g. B?D) was more similar to that of pairs following it (e.g. C?E) than that of pairs preceding it (e.g. A?C), regardless of the pair type. The direction effect was more prominent in new pairs. Again, new pairs were more similar than old pairs in the inferior parietal representation.

The third ANOVA confirmed the direction effect across pair types (Fig. 4E), showing the main effects of Direction (*F*(1,29) = 55.79, *P* < 0.001, *η_p_*^2^ = 0.66) and Hemisphere (*F*(1,29) = 6.86, *P* = 0.01, *η_p_*^2^ = 0.19), and interaction of Direction and Hemisphere (*F*(1,29) = 4.76, *P* = 0.04, *η_p_*^2^ = 0.14). The direction effect was more prominent in the left hemisphere.

## Experiment 2: discrimination task

### Materials and methods

#### Participants

Thirty-one healthy adults participated in experiment 2 (16 women, age 24.1 ± 1.7 years, education 17.1 ± 1.7 years). Inclusion and exclusion criteria were similar to experiment 1. None of the participants had taken part in experiment 1. Six additional participants were excluded due to excessive head motion.

#### Experimental design and procedure

The discrimination task was similar to the transitive inference task, except that all probes were new pairs (50–60 min, Fig. 1B). Participants were asked to judge whether the given relationship between items was true or false following the premises. Participants responded by pressing true/false buttons with their right hand. The true/false button mapping was counterbalanced across participants. No feedback was given. Each participant completed eight experimental blocks, 16 trials per block (eight true pairs, eight false pairs).

#### Statistical analysis of discrimination task performance

We controlled behavioral data quality as in experiment 1. Participants made no premature responses and very few inattentive responses (0.5 ± 0.6%). The inattentive responses were excluded from further analysis.

First, we calculated the discriminability *d*′ of true and false pairs. Second, we examined whether the accuracy was modulated by discrimination using a paired-sample *t*-test (true versus false pairs, *P* < 0.05). Third, we examined whether the RT was modulated by discrimination and serial position using an ANOVA with two factors (*P* < 0.05), Pair (true, false) and Position (AC, BD, CE).

#### Acquisition and preprocessing of MRI and fMRI data

MRI/fMRI data were acquired, preprocessed, and monitored as in experiment 1 (total displacement: all blocks < 1 mm, 0.32 ± 0.10 mm).

#### Univariate analysis of the discrimination and serial position effects

First, we examined whether the inferior parietal activity was modulated by discrimination or serial position. The fourth GLM was built at the subject level, including three true-pair and three false-pair regressors (correct trials only). The premises, incorrect/inattentive trials, and total displacement were included as nuisance regressors. The univariate discrimination contrast was true > false pairs. The univariate serial position contrasts were larger-end > center pairs and smaller-end > center pairs. For true pairs, A > C was the larger end, B > D was the center, and C > E was the smaller end. For false pairs, C > A was the larger end, D > B was the center, and E > C was the smaller end. All contrasts were entered into whole-brain one-sample *t*-tests at the group level (voxel-level *P* < 0.001, cluster-level *P* < 0.05 familywise error correction). In addition, normalized accuracy difference (true > false) was included as a covariate to control possible effects of the accuracy difference on the univariate discrimination contrast.

Second, we visualized the temporal dynamics of the significant univariate discrimination and serial position effects. FIR timeseries of each probe were extracted from ROIs defined by the univariate discrimination and serial position contrasts (see results). For each hemisphere, the percent signal change of 10 scans following the probe (0–15 s) was calculated and entered into paired-sample *t*-tests (discrimination: true > false, serial position: larger end > center, *P* < 0.05 Bonferroni correction).

#### P‌PI analysis of the discrimination effect

First, we examined whether the inferior parietal functional connectivity was modulated by discrimination. Raw fMRI signals of the left (sphere with peak [−48, −33, 21] and 5-voxel radius) and right inferior parietal seeds (sphere with peak [57, −21, 30] and 5-voxel radius) were demeaned and deconvolved to create PPI variables. The fifth GLM was built at the subject level, including a PPI regressor, a physiological signal regressor, and a discrimination contrast regressor. The total displacement was included as a nuisance regressor. The PPI discrimination contrast was defined as true > false pairs and entered into whole-brain one-sample *t*-tests at the group level (voxel-level *P* < 0.001, cluster-level *P* < 0.05 familywise error correction).

Second, we examined whether the accuracy discrimination effect correlated with the left- or right-seed PPI discrimination effect using forward stepwise regression (*P* < 0.05). The accuracy discrimination effect was the mean accuracy difference between true and false pairs, normalized to the accuracy of true pairs.

#### Multivariate decoding analysis of pair type

We examined whether the true/false pairs were decodable from the inferior parietal representation as in experiment 1.

#### Representational similarity analysis of the symbolic distance and direction effects

We examined whether the inferior parietal representational similarity between pairs was modulated by symbolic distance and direction. Figure 6B shows the definition of symbolic distance, direction, and ANOVA. In the fourth GLM, beta values of correct probes were extracted from the left (306 voxels) and right ROIs (308 voxels) defined by the univariate serial position contrast. Pearson correlation coefficients were calculated between pairs, normalized using Fisher’s transformation, and entered into two ANOVAs. The first ANOVA detected the symbolic distance effect with three factors (*P* < 0.05), Distance (1, 2), Pair (true, false), and Hemisphere (left, right). The second ANOVA detected the direction effect with three factors (*P* < 0.05), Direction (preceding/−, following/+), Pair (true, false), and Hemisphere (left, right).

**Fig. 5:**
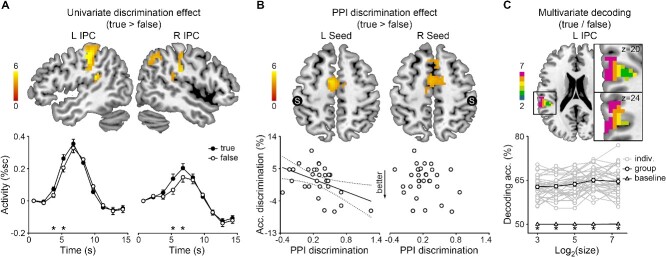
Discrimination effects in the inferior parietal cortex. **A**) Top: Discrimination effects on the regional activity of the left and right inferior parietal cortex (L/R IPC). The color scale indicates *t* values. Bottom: Mean FIR timeseries and SEMs of true and false pairs in the corresponding IPC. %sc, percent signal change; asterisks, *P* < 0.05 corrected. **B**) Top: Discrimination effects on the PPI of the left and right IPC seeds (S). The color scale indicates *t* values. Bottom: The RT discrimination effect correlated with the left-IPC PPI. Solid line, *P* < 0.05; dotted lines, 95% confidence intervals. **C**) Top: Left-IPC ROIs of variable sizes. The color scale indicates log_2_(size). Bottom: Individual data, group means, and SEMs of the decoding accuracy of the left-IPC classifiers. Asterisk, *P* < 0.05 corrected.

**Fig. 6:**
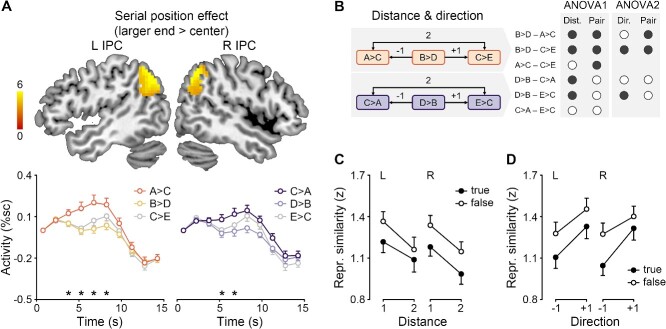
Serial position effect, symbolic distance effect, and direction effect in the inferior parietal cortex. **A**) Top: Serial position effects on the regional activity of the left and right inferior parietal cortex (L/R IPC). The color scale indicates *t* values. Bottom: Mean FIR timeseries and SEMs of true/false pairs in the left IPC. %sc, percent signal change; asterisks, *P* < 0.05 corrected. **B**) Definitions of the symbolic distance, direction, and ANOVA. The hemisphere factor is not shown. Mean inferior parietal representational similarity and SEMs with variable **C**) symbolic distance (ANOVA 1) and **D**) direction (ANOVA 2).

### Results

#### Discrimination task performance

Participants showed a high discriminability of true and false pairs (*d*′ = 3.64 ± 0.62). The accuracy of true pairs was higher than that of false pairs (paired-sample *t*-test, *t*(30) = 2.28, *P* = 0.03, Fig. 1E).

Participants were faster for true than false pairs. The RT increased as the serial position changed, especially in true pairs. The ANOVA revealed main effects of Pair (*F*(1,30) = 41.53, *P* < 0.001, *η_p_*^2^ = 0.58) and Position (*F*(2,60) = 46.73, *P* < 0.001, *η_p_*^2^ = 0.61), and interaction of Pair and Position (*F*(2,60) = 19.40, *P* < 0.001, *η_p_*^2^ = 0.39).

#### Discrimination effects on inferior parietal activity

The inferior parietal activity, but not the hippocampal or lateral prefrontal activity, was modulated by discrimination (Fig. 5A, Supplemental Fig. S1 and Fig. S3). The bilateral inferior parietal regions were more activated for true than false pairs (whole-brain one-sample *t*-test, left: peak [−48, −33, 21], *t* = 6.49, 158 voxels, right: peak [57, −21, 30], *t* = 5.65, 254 voxels). The univariate discrimination effect occurred between 4.5 and 6 s following the probe in the left hemisphere (paired-sample *t*-test, *t*(30) = 3.44–4.41, *P*s < 0.001) and between 6 and 7.5 s in the right hemisphere (*t*(30) = 3.36–4.38, *P*s < 0.001). A similar tendency was obtained on the bilateral inferior parietal ROIs of experiment 1 (Supplemental Fig. S4).

#### Discrimination effects on inferior parietal functional connectivity

The inferior parietal functional connectivity was modulated by discrimination (Fig. 5B). The PPI between the left inferior parietal seed and supplementary motor area (whole-brain one-sample *t*-test, peak [−6, −3, 54], *t* = 5.46, 221 voxels) and that between the right inferior parietal seed and supplementary motor area were enhanced for true than false pairs (peak [−9, −12, 48], *t* = 4.80, 168 voxels).

The normalized accuracy discrimination effect correlated with the left-seed PPI discrimination effect. The forward stepwise regression model for the accuracy discrimination effect (*F*(1,30) = 7.52, *P* = 0.01, *R*^2^ = 0.21) included the left-seed (*t* = −2.74, *P* = 0.01) but not the right-seed PPI discrimination effect (*P* = 0.82). A similar correlation was obtained between the discrimination effects on RT and left-seed PPI (*r* = −0.39, *P* = 0.03).

#### Inferior parietal representation of true/false pairs

The true/false pairs were successfully decoded from the inferior parietal representation (Fig. 5C). The decoding accuracy of the inferior parietal classifier was above baseline regardless of the ROI size and hemisphere (left: 8–161 voxels, *t*(30) = 16.26–23.26, *P*s < 0.001, right: 8–262 voxels, *t*(30) = 16.09–18.92, *P*s < 0.001).

#### Serial position effects on inferior parietal activity

We replicated the finding from experiment 1 that the inferior parietal activity was modulated by serial position (Fig. 6A, Supplemental Fig. S1). The bilateral inferior parietal regions were more activated for true/false pairs near the larger end than those near the center of the series (whole-brain one-sample *t*-test, left: peak [−42, −69, 45], *t* = 8.09, 306 voxels, right: peak [45, −66, 39], *t* = 7.61, 308 voxels). For true pairs, the univariate serial position effect occurred between 4.5 and 9 s following the probe in the left hemisphere (paired-sample *t*-test, *t*(30) = 3.42–5.39, *P*s < 0.001) and between 6 and 7.5 s in the right hemisphere (*t*(30) = 4.27–5.73, *P*s < 0.001). A similar pattern was obtained for false pairs (left: *t*(30) = 3.41–3.65, *P*s < 0.001, right: *t*(30) = 3.03–3.49, *P*s < 0.002). No significant effect was obtained for the smaller end > center contrast.

#### Symbolic distance and direction effects on inferior parietal representations

We replicated the finding from experiment 1 that the inferior parietal representational similarity between pairs was modulated by symbolic distance and direction. The first ANOVA confirmed the symbolic distance effect (Fig. 6C), showing the main effects of Distance (*F*(1,30) = 49.72, *P* < 0.001, *η_p_*^2^ = 0.62) and Pair (*F*(1,30) = 5.54, *P* = 0.02, *η_p_*^2^ = 0.16), but no interaction (*P* = 0.60). No Hemisphere effect was obtained (*P*s > 0.35). The inferior parietal representation of a given pair was more similar to that of pairs adjacent to it than that of pairs distant from it, regardless of the pair type.

The second ANOVA confirmed the direction effect (Fig. 5D), showing the main effects of Direction (*F*(1,30) = 21.68, *P* < 0.001, *η_p_*^2^ = 0.42) and Pair (*F*(1,30) = 7.51, *P* = 0.01, *η_p_*^2^ = 0.20), but no interaction (*P* = 0.32). No Hemisphere effect was obtained (*P*s > 0.31). The inferior parietal representation of a given pair was more similar to that of pairs following it than that of pairs preceding it, regardless of the pair type.

## Discussion

This study investigated the functional role of the inferior parietal cortex in explicit transitive inference. Healthy adults effectively inferred the relationship between a new pair of items they had never observed and discriminated between true and false relationships for a given new pair of items following the premises. First, the inferior parietal cortex, but not the hippocampus or lateral prefrontal cortex, was associated with transitive inference. The inferior parietal functional connectivity was enhanced for new compared to old pairs, although the inferior parietal activity was reduced (inference effect). The inferior parietal activity and functional connectivity were both enhanced for true compared to false pairs (discrimination effect). Moreover, the new/old and true/false pairs were decodable from the inferior parietal representation. Second, the inferior parietal cortex showed three signature effects reflecting the construction of the ordered and directed series. Namely, the inferior parietal activity was enhanced for new/true pairs near the larger end compared to those near the center of the series (serial position effect). The inferior parietal representation of a given new/true pair was more similar to that of new/true pairs adjacent to it than that of new/true pairs distant from it in the series (symbolic distance effect). Finally, the inferior parietal representation of a given new/true pair was more similar to that of new/true pairs following it than that of new/true pairs preceding it in the series (direction effect). These effects suggest that the inferior parietal cortex flexibly integrates observed relations into a relational structure and uses the relational structure to infer unobserved relations.

### Explicit and implicit transitive inference

In this study, we conceptualized and measured transitive inference as an explicit logical reasoning process relying on working memory. Participants would not be able to construct a relational structure at a second-to-minute timescale or use the relational structure to make inferences without the ability to maintain and manipulate information in working memory. The corresponding activations are located at the dorsal part of the inferior parietal cortex associated with working memory demands rather than the ventral part associated with information types (Ravizza et al. 2004).

In previous studies, however, transitive inference has often been conceptualized as an implicit process relying on reinforcement learning (Bryant and Trabasso 1971; McGonigle and Chalmers 1977; Vasconcelos 2008). In the learning stage of a typical implicit transitive inference task, participants are asked to discriminate successive pairs of items in which one item is reinforced and the other is not (e.g. A + B−, B + C−, C + D−, D + E−, where + and − indicate reinforced and unreinforced items, respectively). Participants are expected to learn a relational structure by trial and error (e.g. A > B > C > D > E). In the testing stage, participants view new pairs of items that they have never observed in learning (e.g. B versus D). Choosing B over D is considered a transitive-inference-like behavior.

Transitive-inference-like behavior based on reinforcement learning has been shown in diverse animal species, from fishes to lemurs (von Fersen et al. 1991; Dusek and Eichenbaum 1997; Paz-Y-Miño et al. 2004; Grosenick et al. 2007; Tromp et al. 2015). A distributed brain network comprising the hippocampus, lateral prefrontal cortex, and parietal cortex has been associated with implicit transitive inference. In the learning stage, the hippocampal and parietal activity increases gradually with reinforcement learning (Van Opstal et al. 2008; Van Opstal et al. 2009). In the testing stage, the lateral prefrontal activity increases for inferring unobserved relations of items than for comparing their visual features (Acuna et al. 2002). The hippocampal activity increases for inferring unobserved relations of distant items compared to adjacent items (Zalesak and Heckers 2009). In contrast, the parietal activity decreases for inferring unobserved relations of items than for recalling observed relations of items (Hinton et al. 2010). However, negative evidence exists. Selective lesions to the hippocampus or lateral prefrontal cortex do not necessarily impair transitive-inference-like behavior in humans and animals (Strasser et al. 2004; Frank et al. 2006; Basile et al. 2020).

Frank et al. (2005, 2006) questioned the assumption that transitive-inference-like behavior in animals and humans uses the same system as human explicit logical reasoning. First, they showed that humans could exhibit transitive-inference-like behavior without explicit awareness of the underlying relational structure. The implicit transitive inference performance was simply based on subtle differences in the between-item associative strength developing in reinforcement learning. Second, they showed that the benzodiazepine midazolam could impair the hippocampal explicit memory system while enhancing the implicit transitive inference performance in humans. Third, Ellenbogen et al. (2007) showed that an offline delay of 12–24 h is needed to develop implicit transitive inference from reinforcement learning. In contrast, explicit transitive inference in the current study required minimal time delay. These findings suggest that explicit and implicit transitive inference are dissociable at behavioral and neural levels.

### Inferior parietal activity and functional connectivity underlying transitive inference

Our observation of inference-related parietal activity reduction was similar to previous findings despite the difference in explicit and implicit transitive inference tasks (Hinton et al. 2010). The decreased parietal activity might be a symbolic distance effect. As Hinton et al. showed, the inferior parietal activity was greater for recalling relations of adjacent items (e.g. B > C) than inferring relations of one-step distant items (e.g. B > D), and for inferring relations of one-step than two-step distant items (e.g. B > E). This observation could not be attributed to a familiarity/repetition effect, even though old pairs were presented twice (once as a premise and once as a probe) and new pairs were only presented once. It has been shown that the inferior parietal activity decreases (not increases) for familiar words compared to unfamiliar pseudowords (Taylor et al. 2013) and as the repetition of words and pseudowords increases (Buchsbaum et al. 2015).

Observing different parietal functional connectivity profiles across experiments was not unexpected, as the inference and discrimination contrast isolated distinct cognitive processes. The inference contrast highlighted the application of the transitive law and the retrieval of new information from the relational structure. The superior parietal activity is not directly related to the inference process (Zhang et al. 2022). However, it has been shown to increase for inferring hierarchical (e.g. A > B) compared to equality relations (e.g. A–B) (Wendelken and Bunge 2010) and if hierarchical relations were encoded and retrieved differently (e.g. learning the “larger than” relation but testing the “smaller than” relation) (Hinton et al. 2010). The superior parietal cortex might be engaged to integrate and maintain a hieratical structure in working memory. Enhanced functional connectivity between the superior and inferior parietal cortex might reflect the access to the relational structure, which was critical for new more than old pairs.

The discrimination contrast highlighted the match versus mismatch between the given relationship and the relational structure while keeping the inference process consistent. The supplementary motor area activity appeared in a recent meta-analysis of transitive inference studies (Zhang et al. 2022). However, it might not directly relate to the inference process (Acuna et al. 2002; Wing et al. 2021). Given its role in error monitoring and conflict control (Rae et al. 2014; Fu et al. 2019; Dali et al. 2022), enhanced functional connectivity between the supplementary motor area and inferior parietal might reflect the selection of an appropriate action that demands cognitive control.

### Inferior parietal representations of relational structures

Our findings suggest that the inferior parietal cortex can flexibly map observed relations onto an ordered and directed series and use the series for transitive inference. These findings align with recent views on the role of the posterior parietal cortex in structural knowledge learning (Walsh 2003; Summerfield et al. 2020). It is proposed that in the primate brain, posterior parietal neurons encode relative spatial relations of objects in physical space (e.g. a cat is on the roof) and abstract relations of items in conceptual space (e.g. A is greater than B) through projecting high-dimensional inputs onto a low-dimensional manifold. Moreover, the low-dimensional manifold encodes information across domains. In its simplest form, a one-dimensional manifold is a mental number line (Dehaene 2003; Nieder 2016).

Recent studies have shown evidence supporting the domain-general mental number line. In monkeys, inferior parietal neurons can encode abstract numbers across space, time, and modality (Viswanathan and Nieder 2013; Nieder 2016) and represent them in a linearly or logarithmically scaled continuum (Roitman et al. 2007). In humans, numbers presented in different symbolic formats can be cross-decoded from magnetoencephalography (e.g. training the classifier with digits and testing with dice). The human brain can even map the reward probability and arbitrary features of visual images onto a mental number line (Luyckx et al. 2019; Nelli et al. 2023). For example, Luyckx et al. found that the posterior parietal representation of better-rewarded images was similar to that of larger numbers, and the posterior parietal representation of worse-rewarded images was similar to that of smaller numbers. Although existing evidence is mainly from reinforcement-based implicit learning, it is possible that posterior parietal neurons sensitive to numbers can be recruited to explicitly encode the observed “larger than” relations on a mental number line, apply the transitive law, and promote the inference of unobserved “larger than” relations in working memory.

### Absence of activation differences in the hippocampus and lateral prefrontal cortex

We did not observe inference-related or discrimination-related hippocampal or lateral prefrontal activity. This is not unexpected as selective lesions to the hippocampus or lateral prefrontal cortex do not necessarily impair transitive-inference-like behavior in humans and animals (Strasser et al. 2004; Waechter et al. 2013; Basile et al. 2020).

The hippocampus may serve to derive relational structures through trial and error. In humans, Van Opstal et al. found that the hippocampal activity increased gradually with reinforcement learning, but the increased hippocampal activity did not correlate with implicit transitive inference performance (Van Opstal et al. 2008; Van Opstal et al. 2009). In mice, Barron et al. (2020) found that the hippocampus drew a prospective code of associated objects from incremental learning (e.g. X predicts Y, Y predicts Z). During rest, the coactivation of hippocampal neurons in sharp-wave ripples formed a mnemonic shortcut that can be later used for inferring unlearned associations (e.g. X predicts Z). In this study, however, the relational structure was explicitly constructed through working memory manipulation rather than reinforcement learning. This might lead to the absence of hippocampal activity (for more hippocampal models, see Kumaran 2012).

The involvement of the lateral prefrontal cortex may reflect task difficulty or specific working memory manipulation. For example, Acuna et al. (2002) found that the lateral prefrontal cortex was more activated for inferring unobserved relations of items than comparing their visual features. In addition, Waltz et al. (1999) reported that patients with focal prefrontal lesions made transitive inferences accurately following ordered premises (e.g. A > B, B > C) but not following randomized premises (e.g. B > C, A > B), likely reflecting deficits in reordering premises in working memory (Ye et al. 2020).

### Limitations

This study has limitations. First, it would be ideal to have a longer series that allows us to examine the serial position effect purely with center pairs and the symbolic distance effect at the item level (Vasconcelos 2008; Zalesak and Heckers 2009). For example, given a six-item series (e.g. A > B > C > D > E > F), it should be easier to infer the relation of two-step (e.g. B > E) than one-step distant items (e.g. B > D). However, we were concerned that a longer series and increased number of conditions would lead to longer scanning time and excessive head motion.

Second, the current fMRI parameters might not be ideal for capturing hippocampal BOLD signals. An optimal solution is to record local field potentials directly from the hippocampus with stereoelectroencephalography (SEEG). Our SEEG study showed that hippocampal theta oscillations support the encoding and updating of given visual series in working memory (Su et al. 2024). Hippocampal theta oscillations might also support the construction of new series in working memory (Backus et al. 2016).

Third, we did not examine different strategies used to construct the relational structure. Participants reported their strategies after the transitive inference task in a pilot study. Half of them used a spatial strategy in which they imagined a mental line from the largest to the smallest color. The other half used a verbal strategy like inner speech. The two subgroups performed equally well in the pilot study. Therefore, we did not look into the effect of strategy in this study.

## Conclusion

This study shows that the inferior parietal cortex is critical for explicit logical reasoning like transitive inference. The inferior parietal cortex may integrate observed relations of items into a relational structure and use the relational structure to infer unobserved relations of items. First, the inferior parietal cortex distinguished not only between observed and unobserved relations but also between unobserved relations consistent with and those inconsistent with the underlying relational structure regarding regional activity, functional connectivity, and neural representation. Second, the inferior parietal cortex showed the serial position, symbolic distance, and direction effects on regional activity or neural representation, suggesting the construction of the underlying relational structure. Our findings suggest that the inferior parietal cortex can encode abstract relations on a mental number line to promote generalization.

## Author contributions

Biman Xu (Investigation), Jing Wu (Investigation), Haoyun Xiao (Investigation), Thomas F. Münte (Conceptualization, Writing—review & editing), Zheng Ye (Conceptualization, Formal analysis, Funding acquisition, Visualization, Writing—original draft).

## Funding

This work was supported by the STI2030 Major Projects (2021ZD0203600) and the National Natural Science Foundation of China (31961133025).

*Conflict of interest statement*: None declared.

## Supplementary Material

Suppl_cc240315_bhae137
