# Supplementary material for: Inferior parietal cortex represents relational structures for explicit transitive inference
Source: Cereb Cortex. 2024 Apr 5;34(4):bhae137. doi: 10.1093/cercor/bhae137 (PMC10999362; doi:10.1093/cercor/bhae137)
Supplement: Suppl_cc240315_bhae137 [file suppl_cc240315_bhae137.docx]

Supplemetal Information for

Inferior parietal cortex represents relational structures for explicit transitive inference

Biman Xu,^1,2^ Jing Wu,^1,2^ Haoyun Xiao, ^1,2^ Thomas F. Münte,^3^ and Zheng Ye^1^

^1^ *Institute of Neuroscience, Center for Excellence in Brain Science and Intelligence Technology, Chinese Academy of Sciences, Shanghai 200031, China*

^2^ *University of Chinese Academy of Sciences, Beijing 100049, China*

^3^ *Center for Brain, Behavior & Metabolism, University of Lübeck, Lübeck 23538, Germany*

Correspondence to Z. Ye ([yez@ion.ac.cn](mailto:yez@ion.ac.cn))

1. Experiments 1&2: Results corrected with threshold-free cluster enhancement

We applied the threshold-free cluster enhancement (TFCE) using the pTFCE toolbox (<https://github.com/spisakt/pTFCE>) (Spisák et al., 2019) to double-check the univariate inference, discrimination, and serial position effects (Han et al., 2019; Smith & Nichols, 2009). The pTFCE enhanced image integrates the cluster- and voxel-level information. Therefore, it uses the voxel-level threshold (*p*<0.05 corrected) without specifying any cluster-level threshold. Results corrected with pTFCE were essentially similar to those corrected with SPM12, except that the inference and discrimination effects were left-lateralized.

SFig.1: pTFCE-enhanced (A) inference and serial position effects in Experiment 1, and (B) discrimination and serial position effects in Experiment 2. The color scales indicate adjusted *t* values. IPC, inferior parietal cortex.


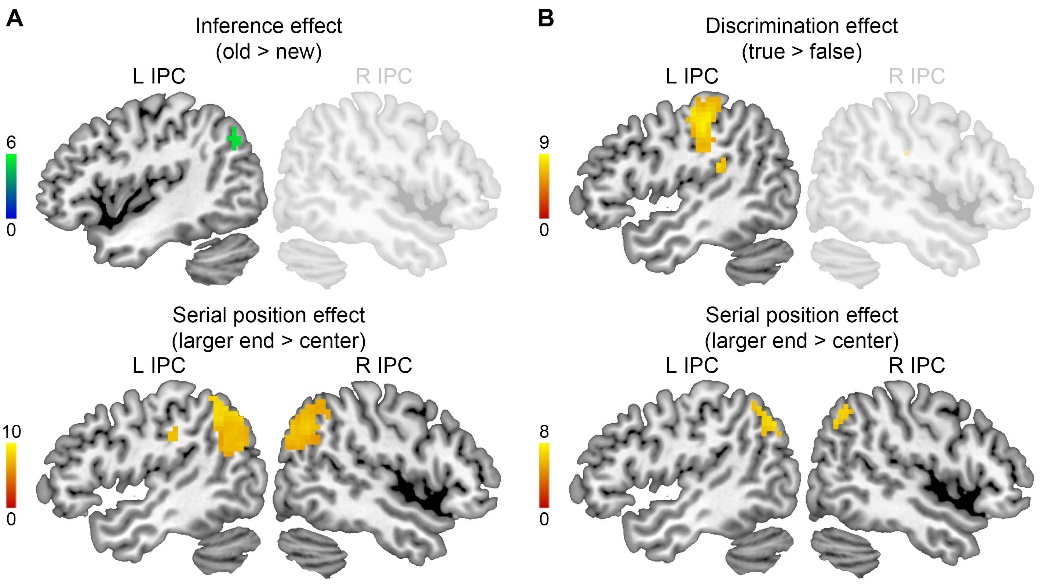


1. Experiment 1: No univariate inference effect in the hippocampus or lateral prefrontal cortex

We found no univariate inference effect in the hippocampus or lateral prefrontal cortex with the whole-brain analysis. Therefore, we applied the ROI analysis to double-check the hippocampal and lateral prefrontal activity. FIR timecourses of each probe were extracted from the anterior and posterior hippocampus (SPM Anatomy toolbox) (Eickhoff et al., 2005) and dorsolateral and ventrolateral prefrontal cortex (AAL atlas). For each subregion and hemisphere, the percent signal change of ten scans following the probe (0-15 s) was calculated and entered into paired-sample *t*-tests (old>new, *p*<0.05 Bonferroni correction). The ROI analysis confirmed the whole-brain analysis: neither the hippocampal nor lateral prefrontal activity was modulated by inference.

SFig.2: (A) Subregions of the hippocampus and lateral prefrontal cortex (PFC). Mean FIR timecourses and SEMs of new and old pairs in (B) the hippocampus and (C) lateral PFC in the left and right hemispheres (L/R). %sc, percent signal change.


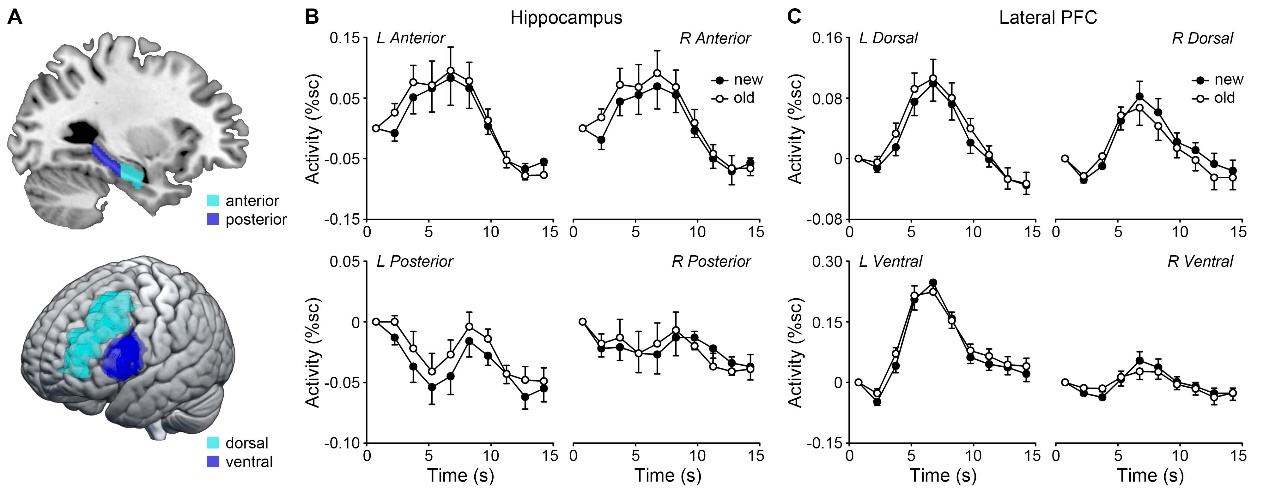


1. Experiment 2: No univariate discrimination effect in the hippocampus or lateral prefrontal cortex

We found no univariate discrimination effect in the hippocampus or lateral prefrontal cortex with the whole-brain analysis. Therefore, we applied the ROI analyisis to double-check the hippocampal and lateral prefrontal activity as in Experiment 1. The ROI analysis confirmed the whole-brain analysis: neither the hippocampal nor lateral prefrontal activity was significantly modulated by discrimination (true>false, *p*<0.05 Bonferroni correction).

SFig.3: (A) Subregions of the hippocampus and lateral prefrontal cortex (PFC). Mean FIR timecourses and SEMs of true and false pairs in (B) the hippocampus and (C) lateral PFC in the left and right hemispheres (L/R). %sc, percent signal change; grey asterisks, *p*<0.05 uncorrected.


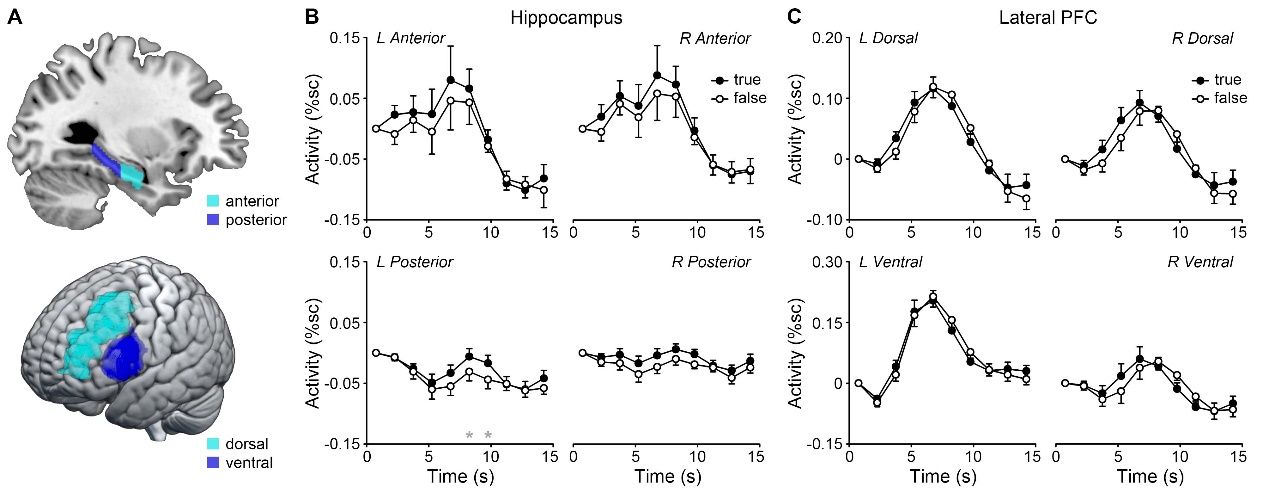


1. Experiment 1: More multivariate decoding analysis and representational similarity analysis

We found no univariate inference effect in the hippocampus, lateral prefrontal cortex, or any other brain regions. Nevertheless, we applied the multivariate decoding analysis (new/old pairs) and representational similarity analysis to the left hippocampus (SPM Anatomy toolbox, 141 voxels) and lateral prefrontal cortex (AAL atlas, 1567 voxels). The multivariate decoding analysis confirmed the univariate analysis: the new/old pairs were not correctly decoded from the hippocampus or lateral prefrontal cortex. Only the lateral prefrontal cortex showed the direction effect.

STable 1: Multivariate decoding analysis

| Region | Mean±SD (%) | One-sample *t* test (*p* value) |
| --- | --- | --- |
| Left hippocampus | 51.5±10.3 | 0.44 |
| Left lateral prefrontal cortex | 42.7±1.0 | <0.001 (opposite) |

Stable 2: Representational similarity analysis

| Region | ANOVA1 (*p* value) | | | ANOVA2 (*p* value) | | |
| --- | --- | --- | --- | --- | --- | --- |
|  | Distance | Pair | Distance*Pair | Direction | Pair | Direction*Pair |
| Left hippocampus | <0.001 | 0.002 | 0.005 | 0.807 | <0.001 | 0.161 |
| Left lateral prefrontal cortex | <0.001 | 0.001 | 0.090 | <0.001 | <0.001 | 0.612 |

1. Experiment 2: Univariate discrimination effects in the inferior parietal ROIs from Experiment 1

We examined whether the inferior parietal regions showing the univariate inference effect also showed the univariate discrimination effect. In Experiment 2, FIR timecourses of each probe were extracted from the inferior parietal ROIs defined by the univariate inference contrast of Experiment 1. For each hemisphere, the percent signal change of ten scans following the probe (0-15 s) was calculated and entered into paired-sample *t*-tests (true>false, *p*<0.05).

The inferior parietal ROIs showing the inference effect in Experiment 1 tended to show the discrimination effect in Experiment 2. The marginal discrimination effect occurred between 4.5-7.5 s following the probe in the left hemisphere and between 6-9 s in the right hemisphere. Although the effects did not pass a Bonferroni correction (possiblely due to different samples), it suggests that both inference and discrimination modulated the inferior parietal activity.

SFig.4: Mean FIR timecourses and SEMs of true and false pairs in the left and right inferior parietal cortex (L/R IPC). %sc, percent signal change; grey asterisks, *p*<0.05 uncorrected.


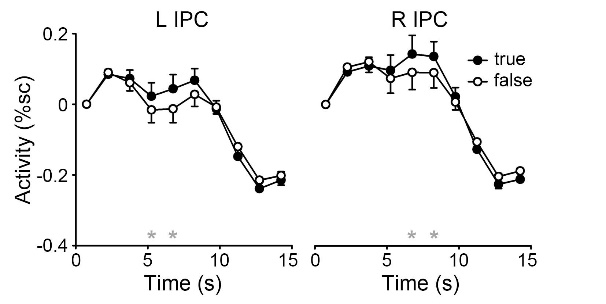


References

Eickhoff, S. B., Stephan, K. E., Mohlberg, H., Grefkes, C., Fink, G. R., Amunts, K., & Zilles, K. (2005). A new SPM toolbox for combining probabilistic cytoarchitectonic maps and functional imaging data. *Neuroimage, 25*(4), 1325-1335.

Han, H., Glenn, A. L., & Dawson, K. J. (2019). Evaluating alternative correction methods for multiple comparison in functional neuroimaging research *Brain Sciences, 9*(8), 198.

Smith, E. E., & Nichols, T. E. (2009). Threshold-free cluster enhancement: Addressing problems of smoothing, threshold dependence and localisation in cluster inference. *Neuroimage, 44*, 83-98.

Spisák, T., Spisák, Z., Zunhammer, M., Bingel, U., Smith, S., Nichols, T., & Kincses, T. (2019). Probabilistic TFCE: A generalized combination of cluster size and voxel intensity to increase statistical power. *Neuroimage, 185*, 12-26.

Zhang, X., Qiu, Y., Li, J., Jia, C., Liao, J., Chen, K., Qiu, L., Yuan, Z., & Huang, R. (2022). Neural correlates of transitive inference: An SDM meta-analysis on 32 fMRI studies. *Neuroimage, 258*, 119354.
